# Clinical significance of the controlling nutritional status (CONUT) score in gastric cancer patients: A meta-analysis of 9,764 participants

**DOI:** 10.3389/fnut.2023.1156006

**Published:** 2023-04-11

**Authors:** Hui Liu, Xiao-Chuan Yang, Ding-Cheng Liu, Chao Tong, Wen Wen, Ri-Hui Chen

**Affiliations:** ^1^Department of Interventional Radiology, Xiangya School of Medicine Affiliated Haikou Hospital, Central South University, Haikou, Hainan, China; ^2^Department of Hepatobiliary Surgery, Xiangya School of Medicine Affiliated Haikou Hospital, Central South University, Haikou, Hainan, China

**Keywords:** controlling nutritional status score, postoperative complications, survival outcomes, meta-analysis, gastric cancer

## Abstract

**Background:**

The clinical value of the controlling nutritional status (CONUT) score has been widely reported in multiple malignancies. The aim of this study is to investigate the association between the CONUT score and clinical outcomes in patients with gastric cancer.

**Methods:**

A comprehensive literature search of electronic databases including PubMed, Embase, and Web of Science was performed up to December 2022. The primary endpoints were survival outcomes and postoperative complications. Subgroup analysis and sensitivity analysis were performed during the pooled analysis.

**Results:**

Nineteen studies including 9,764 patients were included. The pooled results indicated that patients in the high CONUT group had a worse overall survival (HR = 1.70 95%CI: 1.54–1.87; *P* < 0.0001; *I*^2^ = 33%) and recurrence-free survival (HR = 1.57; 95%CI: 1.36–1.82; *P* < 0.0001; *I*^2^ = 30%), and a higher risk of complications (OR = 1.96; 95%CI: 1.50–2.57; *P* < 0.0001; *I*^2^ = 69%). In addition, a high CONUT score was significantly associated with larger tumor size, higher percentage of microvascular invasion, later TNM stage and fewer patients receiving adjuvant chemotherapy, but not with tumor differentiation.

**Conclusion:**

Based on existing evidence, the CONUT score could act as a valuable biomarker to predict clinical outcomes in patients with gastric cancer. Clinicians could use this useful indicator to stratify patients and formulate individual treatment plans.

## 1. Background

Gastric cancer (GC) remains the fifth most frequently diagnosed cancer and the third leading cause of cancer-related deaths in the world (1, 2). Despite advances in perioperative therapies and surgical techniques for GC patients, the clinical prognosis for GC has not significantly improved until now, mainly due to early recurrence and metastasis (3, 4). It is important to formulate treatment plans based on the expected survival time of patients to improve the cure rate for GC. Currently, the treatment of GC is mainly based on the AJCC TNM staging system. However, the staging system alone does not support treatment selection and prognosis assessment of GC well (5, 6). Therefore, it is essential to explore novel prognostic biomarkers to guide treatment of GC.

As indicated by growing evidence, host’s nutrition status plays a critical role in the progression and survival of cancer patients (7). Based on these insights, several nutritional indicators have been successfully constructed to predict outcomes in cancer patients (8, 9). Among these, the controlling nutritional status (CONUT) score, which is calculated using peripheral albumin level, total cholesterol level and total lymphocyte count, has been developed as a nutritional screening tool (Table 1) (10). Recently, the clinical value of the CONUT score for predicting short-term and long-term outcomes has been widely reported in solid tumors and hematologic malignancies (11). The impact of the CONUT score on outcomes in GC patients was first reported in 2017 (12). After that, a growing number of studies have further explored the relationship between the CONUT score and clinical outcomes in GC patients (13–18). In 2019, Takagi et al. (19) preliminarily confirmed the prognostic value of CONUT score in GC by pooling five studies. Nevertheless, the authors acknowledge that the included studies are limited, and the role of the CONUT score in GC patients is actually unclear. Given that additional reports have been published in recent years, we therefore performed a meta-analysis based on available evidence to further investigate the association between the CONUT score and outcomes in patients with GC.

**TABLE 1:** The scoring criteria for the CONUT score.

Variables	Degree			
	**Normal**	**Mild**	**Moderate**	**Severe**
**Albumin level (g/dl)**	≥3.50	3.00–3.49	2.50–2.99	<2.50
Score	0	2	4	6
**Cholesterol level (mg/dl)**	≥1,600	1,200–1,599	800–1,199	<800
Score	0	1	2	3
**Total lymphocyte count (/ml)**	≥180	140–179	100–139	<100
Score	0	1	2	3
**CONUT score**	0–1	2–4	5–8	9–12

## 2. Methods

### 2.1. Search strategy

The current study was conducted according to the Preferred Reporting Items for Systematic Reviews and Meta-Analyses (PRISMA) guidelines to identify studies that assess the association of the CONUT score with clinical outcomes in GC patients. Relevant studies from PubMed, Embase, and Web of Science were comprehensively examined up to 1 December 2022. The following combination of key words was used to search the potential related studies: (“CONUT”) AND (“gastric cancer” OR “stomach cancer” OR “stomach tumor”). Language restriction was not applied during the search process. In addition, the references of the included studies were further scanned for extra reports. The search was independently performed by two investigators (HL and X-CY).

### 2.2. Study selection

The inclusion criteria were presented as follows: (1) studies examined the relationship between the CONUT score and clinical outcomes of GC patients; (2) the outcomes including survival outcomes and/or complications were available; (3) the cut-off value of the CONUT was clearly reported; and (4) studies were published in any language.

The exclusion criteria were as follows: (1) studies did not report data for GC patients separately; (2) studies were reported as case reports, reviews, conferences and letters; (3) duplicated data; and (4) studies was not peer reviewed.

### 2.3. Data extraction and quality assessment

Two reviewers (HL and X-CY) conducted the data extraction independently and cross-checked all the results. The extracted data included first author, publication year, study interval, country, study design and sample size, selection method, cut-off value, clinicopathological features like age, sex, tumor size, tumor differentiation, microvascular invasion, tumor stage and adjuvant chemotherapy, and clinical outcomes including postoperative complications, survival data, and follow-up time. When necessary, the authors would be contacted to provide relevant data.

The quality assessment of included studies was performed following the method described by Lin et al. (20) with the following nine items: (1) clear description of purpose/objectives, (2) clear ethical statements, (3) clear description of tumor stage and/or clinical setting, (4) clear description of inclusion criteria, (5) clear description of the cutoff value, (6) predefinition of outcome measurements, (7) whether or not use multivariate analysis and/or univariate analysis, (8) long enough follow-up period, and (9) limitations considered. Finally, a study could get a final score from 0 to –9 after assessment. Quality assessment was not used as exclusion criterion for these 19 included studies.

### 2.4. Outcomes assessment

In this study, the primary outcomes were postoperative complications and survival outcomes including overall survival (OS), recurrence-free survival (RFS), progression-free survival (PFS), disease-free survival (DFS), and cancer-specific survival (CSS). The postoperative complications were defined as any morbidities occurred with 30 days after gastrectomy and graded by Clavien-Dindo (CD) (21, 22) system. Since RFS, PFS, DFS, and CSS share similar endpoints, they were analyzed together as one outcome, RFS, as previously suggested (23, 24). The secondary outcomes were other postoperative oncological parameters, including tumor size (<5 cm), tumor differentiation (poor differentiation), and TNM stage (Stage III/IV), microvascular invasion (Yes), and adjuvant chemotherapy (Yes).

### 2.5. Statistical analysis

The odds ratios (ORs) and hazard ratios (HRs) with their 95% confidence intervals (CIs) were used as the effect size for postoperative complications and survival outcomes, respectively. Statistical heterogeneity among enrolled studies was assessed using *I*^2^ statistic. When *I*^2^ is less than 50%, a fixed-effect model was used to calculate the pooled estimates; otherwise, a random-effects model was performed. Subgroup analysis and sensitivity analysis were utilized to evaluate the credibility of pooled results. Begg’s funnel plot was applied to assess the possibility of publication bias. A two-tailed *P*-value <0.05 was considered statistically significant. All of these statistical analyses were performed by Review Manager Software, version 5.3 (Cochrane, London, UK) and Stata, version 12.0 (Statacorp, College Station, TX, USA).

## 3. Results

### 3.1. Study characteristics

**FIGURE 1:**
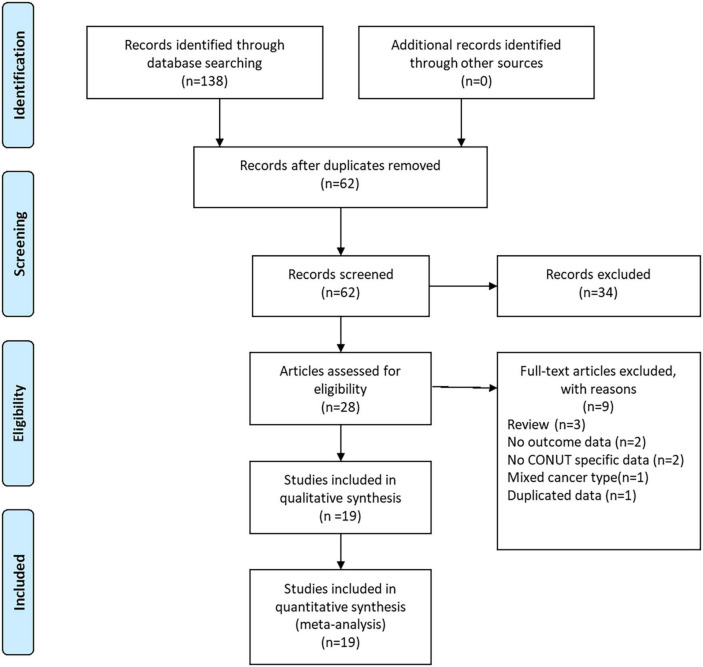
The PRISMA flowchart of study selection.

As shown in Figure 1, a total of 138 records were yielded after searching the databases. Through careful title, abstract assessment and full text assessment, 19 studies (12–18, 25–36) with 21 cohorts were finally included in the present study. The basic information and clinical characteristics of the included studies were summarized in Tables 2, 3, respectively. A total of 9,764 patients from China, Japan, Korea, and Turkey were included in this study. These studies were published from 2017 to 2022 with a sample size ranging from 33 to 2182. In terms of primary treatment, surgery was performed in 16 studies, neoadjuvant therapy was performed in two studies and mixed treatment including immunotherapy was performed in one study. The quality of the included studies was good with a median score of 9 (range: 6–9, Figure 2 and Supplementary Table 1).

**TABLE 2:** Basic information of included studies.

Author	Publication year	Country	Study design	Study interval	Sample size	Age, years	Sex (male/female)	TNM stage
Akagunduz	2021	Turkey	R; S	2017–2021	161	58.7 (range,32–80)	110/51	I–III
Aoyama	2022	Japan	R; S	2013–2017	331	NA	219/112	I–III
Chen	2022	China	R; S	2016–2020	146	59 (range,34–82)	102/44	I–IV
Hirahara	2019	Japan	R; S	2010–2016	210	NA	146/64	I–III
Huang	2019	China	P; S	2014–2016	357	73.29 ± 5.24	275/82	I–III
Jeon	2020	Korea	R; S	2009–2015	1,307	NA	862/445	I–III
Jin	2021	China	R; S	2004–2015	272	61 (range, 32–80)	201/71	0–III
Kudou	2019	Japan	R; S	2005–2016	144	65 (range,35–91)	104/40	I–III
Kuroda	2018	Japan	R; S	2005–2014	416	67.2 (range 25–94)	276/149	I–III
Lin	2019	China	R; S	2009–2014	2,182	60.8 (IQR, 54–68.3)	1,643/539	I–III
Liu	2018	China	R; S	2000–2012	697	57 (range, 21–86)	457/230	II–III
Mimatsu	2017	Japan	R; S	2006–2016	33	NA	28/6	IV
Qian	2021	China	R; S	2016–2019	309	63.4 ± 0.6	228/81	I–IV
Ryo	2019	Japan	R; M	2010–2014	626	67.9 ± 10.9	435/191	II–III
Sun	2021	China	R; S	2016–2018	1,479	60.4 ± 17.3	1,083/396	I–IV
Suzuki	2021	Japan	R; S	2000–2015	211	≥75	141/70	I–III
Xiao	2022	China	R; S	2014–2019	106	67 (range,43–85)	84/22	I–IV
Zheng	2018	China	R; S	2010–2011	532	61.1 ± 11.5	403/129	I–III
Zhu	2021	China	R; S	2005–2015	245	NA	179/66	I–IV

R, retrospective; S, single center; M, multiple center; NA, not available; IQR, inter-quartile range; OS, overall survival; PFS, progression-free survival; RFS, recurrence-free survival; DFS, disease-free survival.

**TABLE 3:** Survival information of included studies.

Author	Publication year	Sample size	Low group	High group	Primary treatment	Selection method	Cut-off value	Multivariate analysis	Survival outcomes	Median follow-up time, months
Akagunduz	2021	161	56	105	Neoadjuvant chemotherapy	ROC	≥4	Yes	OS	11.2 (range:2.3–32.3)
Aoyama	2022	331	221	110	Curative surgery	NA	≥2	Yes/Yes	OS; RFS	NA
Chen	2022	146	75	71	PD-1/PD-L1 inhibitors or chemotherapy	NA	>0	Yes/Yes	OS; PFS	NA
Hirahara	2019	210	105	105	Curative surgery	ROC	≥3	Yes	OS	35.3 (range:4.0–97.0)
Huang	2019	357	153	204	Curative surgery	NA	≥2	NA	NA	NA
Jeon	2020	1,307	Normal: 893	Light:396; Moderate:18; Severe:1	Curative surgery	NA	NA	Yes	OS	59.0 (range: 1–109)
Jin	2021	272	182	85	Neoadjuvant chemotherapy	ROC	≥4	Yes/Yes	OS; PFS	NA
Kudou	2019	144	118	26	Curative surgery	ROC	≥3	No/No	OS; RFS	NA
Kuroda	2018	416	354	62	Curative surgery	ROC	≥4	Yes/No	OS; RFS	61.2 (range: 1–134)
Lin	2019	2,182	1704	478	Curative surgery	X-tile	>2	No	OS	52 (range: 1–118)
Liu	2018	697	480	217	Curative surgery	ROC	≥3	Yes	CSS	36 (range: 3–162)
Mimatsu	2017	33	16	17	Non-curative surgery	NA	>4	No	OS	NA
Qian	2021	309	214	95	Curative surgery	ROC	2.5	NA	NA	NA
Ryo	2019	626	337	289	Curative surgery	ROC	≥2	Yes/No	OS; DFS	49.2
Sun	2021	1,479	627	852	Curative surgery	ROC	≥2	NA	NA	NA
Suzuki	2021	211	175	36	Curative surgery	NA	>4	Yes/Yes	OS; CSS	47 (range: 5–185)
Xiao	2022	106	43	63	Curative surgery	NA	>4	No	OS	30 (range:7–64)
Zheng	2018	532	Normal:291	Light: 183; Moderate or severe: 58	Curative surgery	NA	NA	Yes/Yes	OS; RFS	60 (range: 2- 76)
Zhu	2021	245	104	141	Curative surgery	ROC	≥4	Yes/Yes	OS; DFS	NA

ROC, receiver operating characteristic curve; NA, not available; OS, overall survival; PFS, progression-free survival; RFS, recurrence-free survival; DFS, disease-free survival; CSS, cancer specific survival.

**FIGURE 2:**
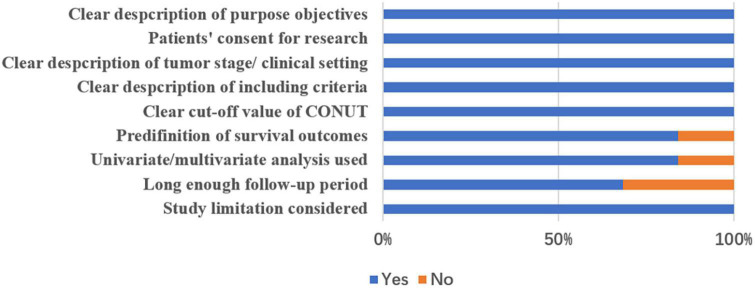
Quality assessment of included studies.

### 3.2. Relationship between the CONUT and OS

**FIGURE 3:**
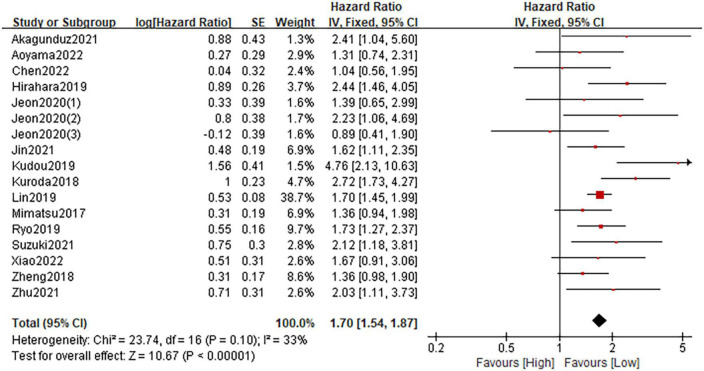
Forest plot assessing the relationship between the CONUT and OS.

Fifteen studies involving 6,922 patients described the association between the CONUT and OS. The fixed-effect model was applied due to the low heterogeneity (*I*^2^ = 33%; *P* = 0.10). The pooled HR was 1.70 (95%CI: 1.54–1.87; *P* < 0.0001), which indicated that a high CONUT score was significantly associated with worse OS in patients with gastric cancer (Figure 3). Furthermore, subgroup analyses based on country, sample size, primary treatment, cut-off method, cut-off value and analysis method were performed. As shown in Table 4 and Supplementary Figure 1, the pooled results from all subgroup analyses revealed that patients in the high CONUT group had a substantially reduced OS when compared to these in the low CONUT group. In addition, sensitivity analysis by omitting one study at a time demonstrated that the combined outcome was not significantly changed (Supplementary Figure 3A).

**TABLE 4:** Subgroup analyses for OS and RFS of CONUT-high patients vs. CONUT-low patients.

		Cohorts, *n*	Patients, *n*	HR (95%CI)	*P*-value	*I*^2^ (%)
**Overall survival**						
	Total	17	6,922	1.70 (1.54–1.87)	<0.0001	33
Country	China	6	3,483	1.62 (1.43–1.83)	<0.0001	0
	Japan	7	1,971	1.90 (1.60–2.26)	<0.0001	56
	Others	4	1,468	1.58 (1.07–2.33)	0.02	26
Sample size	>200	12	6,332	1.72 (1.54–1.91)	<0.0001	16
	≤200	5	590	1.61 (1.25–2.08)	<0.0001	62
Primary treatment	Surgery	14	6,343	1.72 (1.55–1.91)	<0.0001	37
	Others	3	579	1.54 (1.14–2.07)	0.005	25
Cut-off method	ROC	7	2,074	2.06 (1.72–2.46)	<0.0001	33
	Others	10	4,848	1.57 (1.39–1.76)	<0.0001	0
Cut-off value	≥4	7	1,444	1.82 (1.51–2.19)	<0.0001	10
	<4	10	5,478	1.66 (1.48–1.86)	<0.0001	45
Analysis method	Univariate	4	2,465	1.70 (1.48–1.95)	<0.0001	61
	Multivariate	13	4,457	1.70 (1.49–1.95)	<0.0001	25
**Recurrence free survival**						
	Total	10	3,620	1.57 (1.36–1.82)	<0.0001	30
Country	China	5	1,892	1.50 (1.25–1.81)	<0.0001	0
	Japan	5	1,728	1.70 (1.34–2.16)	<0.0001	53
Sample size	>200	8	3,330	1.56 (1.34–1.82)	<0.0001	13
	≤200	2	290	1.69 (1.02–2.79)	0.04	79
Primary treatment	Surgery	8	3,202	1.60 (1.36–1.89)	<0.0001	40
	Others	2	418	1.47 (1.07–2.02)	0.02	0
Cut-off method	ROC	6	2,400	1.64 (1.36–1.97)	<0.0001	39
	Others	4	1,220	1.47 (1.16–1.87)	0.002	29
Cut-off value	≥4	4	1,144	1.99 (1.47–2.70)	<0.0001	24
	<4	6	2,476	1.47 (1.24–1.73)	<0.0001	15
Analysis method	Univariate	3	1,186	1.60 (1.22–2.12)	0.0008	67
	Multivariate	7	2,434	1.56 (1.31–1.86)	<0.0001	10

### 3.3. Relationship between the CONUT and RFS

**FIGURE 4:**
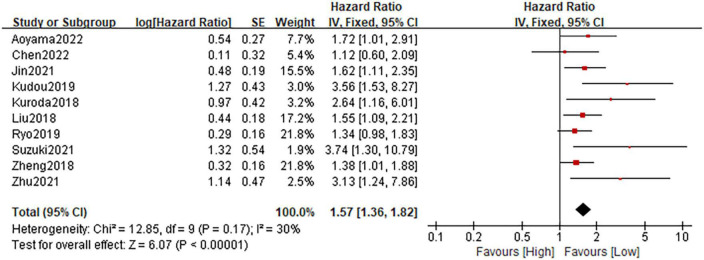
Forest plot accessing the relationship between the CONUT and RFS.

A total of ten studies consisting of 3,620 patients reported on RFS. The heterogeneity test showed a low heterogeneity among studies (*I*^2^ = 30%; *P* = 0.17), and the fixed-effect model was performed. The pooled HR was 1.57 (95%CI: 1.36–1.82; *P* < 0.0001), which suggested that patients in the high CONUT group had a significantly poorer RFS when compared with patients in the low CONUT group (Figure 4). Similarly, Stratification by country, sample size, primary treatment, cut-off method, cut-off value, and analysis method showed that the incorporated results were consistent in each subgroup (Table 4 and Supplementary Figure 2). Sensitivity analysis demonstrated that the pooled result remained unchanged (Supplementary Figure 3B).

### 3.4. Relationship between the CONUT and postoperative complications

**FIGURE 5:**
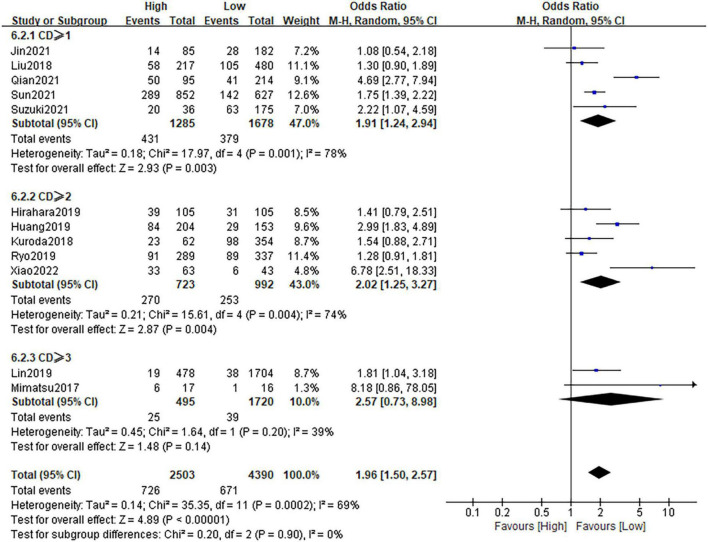
Forest plot assessing the relationship between the CONUT and postoperative complications.

Twelve studies, comprising 6,893 patients, investigated postoperative complications in patients with gastric cancer. Following the result of heterogeneity test (*I*^2^ = 69%; *P* = 0.0002), the random-effect model was applied. The pooled OR was 1.96 (95%CI: 1.50–2.57; *P* < 0.0001), which suggested that a high CONUT score was a risk factor of postoperative complications for gastric cancer patients (Figure 5). Stratification by CD grade showed that the pooled results were almost unchanged in each subgroups. Sensitivity analysis confirmed the credibility of the combined result (Supplementary Figure 3C).

### 3.5. Relationship between the CONUT and other postoperative oncological parameters

As shown in Table 5 and Supplementary Figure 4, the pooled results revealed that a higher CONUT score was associated with larger tumor size (OR = 0.60; 95%CI:0.49–0.75; *P* < 0.0001; *I*^2^ = 0%), higher percentage of microvascular invasion (OR = 0.67; 95%CI:0.50–0.89; *P* = 0.006; *I*^2^ = 0%), later TNM stage (OR = 0.63; 95%CI:0.55–0.72; *P* < 0.0001; *I*^2^ = 35%) and fewer patients receiving adjuvant chemotherapy (OR = 1.44; 95%CI:0.98–2.12; *P* = 0.06; *I*^2^ = 68%). Nevertheless, no significant association was found in tumor differentiation (OR = 1.03; 95%CI:0.88–1.20; *P* = 0.72; *I*^2^ = 33%).

**TABLE 5:** Secondary outcomes in terms of CONUT-high patients vs. CONUT-low patients.

Variables	Cohorts, *n*	Patients, *n*	OR (95%CI)	*P*-value	*I*^2^ (%)
Tumor size (<5 cm)	3	1,469	0.60 (0.49–0.75)	<0.0001	0
Tumor differentiation (Poor)	11	3,544	1.03 (0.88–1.20)	0.72	33
Microvascular invasion (Yes)	3	1,645	0.67 (0.50–0.89)	0.006	0
TNM stage (Stage III/IV)	12	4,122	0.63 (0.55–0.72)	<0.0001	35
Adjuvant chemotherapy (Yes)	6	1,913	1.44 (0.98–2.12)	0.06	68

### 3.6. Publication bias

The Begg’s funnel plots of the primary outcomes were displayed in Supplementary Figure 5. Begg’s test revealed that there was no significant publication bias in the present study about CONUT score and OS (*P* = 0.680), RFS (*P* = 0.602), and postoperative complications (*P* = 0.304).

## 4. Discussion

Malnutrition is common in cancer patients, which is further exacerbated in gastric cancer patients due to additional factors such as malabsorption and obstructive syndrome (37). Numerous pieces of evidence have illustrated that malnutrition can lead to increased length of hospital stays and deteriorate the prognosis of cancer patients (38, 39). Therefore, early screening and proper treatment of malnourished patients is extremely important in clinical practice. Currently, although several tumor-related nutrition assessment tools like NRS2002 and PG-SGA have been developed (40, 41), the utilization of these tools is controversial due to their complexity and subjectivity. Ideally, the screening tool should be simple, convenient, sensitive and objective.

In this context, the CONUT score was constructed by González-Madroño et al. (42) in 2012 as a potential tool to make clinical undernutrition screening using three peripheral blood parameters (albumin level, total cholesterol level, and total lymphocyte count). Since then, the CONUT has been gradually used to assess the prognosis of various cancers due to its easy availability and convenient calculation (43–45). Niu et al. in a meta-analysis of 12 studies have reported that high CONUT score is associated with worse survival OS and CSS in urological cancers (46). Another meta-analysis by Takagi et al. also confirmed that the CONUT score is a practical prognostic factor associated with the prognosis of colorectal cancer (45). Additionally, the clinical value of the CONUT score has been successfully validated in other malignancies, such as lung cancer (47) and hepatocellular carcinoma (48). However, since each cancer type varies a lot, it is important to explore the applicability of the CONUT score in gastric cancer.

We conducted a comprehensive literature search and identified 19 studies with 9,764 GC patients. Relative to previous studies (19), this update has several strengths. First, by including all patients in our study, the generalizability of the CONUT score as a predictive marker in GC patients is enhanced compared to previous studies that only included patients undergoing radical resection. Second, by including an adequate number of samples, the heterogeneity of the pooled survival outcomes is significantly reduced. Third, due to the full inclusion of all studies, we are able to perform adequate subgroup analyses to fully explore the ability of the CONUT score as a nutritional screening metric to predict clinical outcomes in different kinds of GC patients. Through our pooled analyses, we found that patients in the high CONUT score group had 1.70, 1.57, and 1.96 times increased risk of the poor OS and RFS, as well as higher incidence of postoperative complications, compared to those with low CONUT score. Besides, we noted that high COUNT score was significantly associated with larger tumor size, higher percentage of microvascular invasion, later TNM stage and fewer patients receiving adjuvant chemotherapy. On examination of all subgroup analyses, it can be seen that all of the pooled outcomes supported the efficacy of the CONUT score in the primary outcomes prediction. Meanwhile, the pooled outcomes remained their significance on sensitivity analyses, and no evidence of publication bias was observed through Begg’s tests. The results were robust and therefore increase the credibility of our conclusions.

The good discriminatory value of the CONUT score could be explained as follows: Firstly, each of the components of the CONUT score has been demonstrated to be associated with outcomes in cancer patients. Albumin as a recognized indicator has been widely used to reflect a patient’s nutritional status. Hypoalbuminemia has been demonstrated to be significantly associated with poor wound healing, increased risk of infections and reduced survival of cancer patients (49, 50). In addition, serum albumin plays an important role in inhibiting the production of pro-inflammatory cytokines and enhancing cell-mediated immunity (51). And low levels of albumin thereby reduce response to adjuvant therapy. Secondly, cholesterol, as an important component of the cell membrane, plays an essential role in maintaining the cellular function. Low levels of cholesterol have been suggested to prompt tumor progression and deteriorate patient prognosis in various cancers (52, 53). The underlying mechanism may be a consequence of the requirement of cholesterol consumption for tumor growth (54). In addition, a recent study based on animal models showed that high serum cholesterol levels can enhance the antitumor effect of natural killer cells in mice (55). Finally, lymphocyte count, an important indicator of immune and nutritional status in cellular immunity, has been confirmed to inhibit tumor progression by inducing its lysis and apoptosis (56). Numerous studies have demonstrated that lymphopenia is strongly associated with early recurrence and poor survival in cancer patients (57). Secondly, our pooled results further revealed that higher COUNT score was significantly related to larger tumor size, higher percentage of microvascular invasion, later TNM stage and fewer patients receiving adjuvant chemotherapy, even though it remains unclear whether the results of the CONUT score were a cause or a consequence of these advanced tumor characteristics.

The present meta-analysis had several limitations. First, all of these studies were retrospective in nature, which may increase the risk of selection bias, and more prospective studies are thereby required to further investigate this issue. Second, most included studies were from Asian countries, which may affect the applicability of the CONUT score in Western populations. Third, the cut-off value of the CONUT score varies greatly among studies, which might affect the clinical utility of these findings.

## 5. Conclusion

Our results suggested that the CONUT score could be a valuable prognostic biomarker for patients with gastric cancer. Patients in the high CONUT score group have poor OS, RFS, and a higher rate of complications. Clinicians could use this useful indicator to stratify patients and formulate individual treatment plans. However, further research is still required to validate the value of this index in gastric malignancy.

## Author contributions

HL wrote the manuscript. HL and X-CY performed the data search and data analysis. HL, X-CY, and D-CL prepared the figures and tables. WW and R-HC approved the final manuscript. All authors reviewed the manuscript.
