# Supplementary material for: Clinical significance of the controlling nutritional status (CONUT) score in gastric cancer patients: A meta-analysis of 9,764 participants
Source: Front Nutr. 2023 Apr 11;10:1156006. doi: 10.3389/fnut.2023.1156006 (PMC10126262; doi:10.3389/fnut.2023.1156006)
Supplement: Supplementary file 1 [file Data_Sheet_1.docx]

Table S1. Quality assessment of included studies.

| **Items** | **Akagunduz** | **Aoyama** | **Chen** | **Hirahara** | **Huang** | **Jeon** | **Jin** | **Kudou** | **Kuroda** | **Lin** | **Liu** | **Mimatsu** | **Qian** | **Ryo** | **Sun** | **Suzuki** | **Xiao** | **Zheng** | **Zhu** |
| --- | --- | --- | --- | --- | --- | --- | --- | --- | --- | --- | --- | --- | --- | --- | --- | --- | --- | --- | --- |
| **Study limitation considered** | 1 | 1 | 1 | 1 | 1 | 1 | 1 | 1 | 1 | 1 | 1 | 1 | 1 | 1 | 1 | 1 | 1 | 1 | 1 |
| **Long enough follow-up period** | 0 | 1 | 0 | 1 | 0 | 1 | 1 | 1 | 1 | 1 | 1 | 0 | 0 | 1 | 0 | 1 | 1 | 1 | 1 |
| **Univariate/multivariate analysis used** | 1 | 1 | 1 | 1 | 0 | 1 | 1 | 1 | 1 | 1 | 1 | 1 | 0 | 1 | 0 | 1 | 1 | 1 | 1 |
| **Predifinition of OS/RFS** | 1 | 1 | 1 | 1 | 0 | 1 | 1 | 1 | 1 | 1 | 1 | 1 | 0 | 1 | 0 | 1 | 1 | 1 | 1 |
| **Clear cut-off value of CONUT** | 1 | 1 | 1 | 1 | 1 | 1 | 1 | 1 | 1 | 1 | 1 | 1 | 1 | 1 | 1 | 1 | 1 | 1 | 1 |
| **Clear despcription of including criteria** | 1 | 1 | 1 | 1 | 1 | 1 | 1 | 1 | 1 | 1 | 1 | 1 | 1 | 1 | 1 | 1 | 1 | 1 | 1 |
| **Clear despcription of tumor stage/ clinical setting** | 1 | 1 | 1 | 1 | 1 | 1 | 1 | 1 | 1 | 1 | 1 | 1 | 1 | 1 | 1 | 1 | 1 | 1 | 1 |
| **Patients' consent for research** | 1 | 1 | 1 | 1 | 1 | 1 | 1 | 1 | 1 | 1 | 1 | 1 | 1 | 1 | 1 | 1 | 1 | 1 | 1 |
| **Clear despcription of purpose objectives** | 1 | 1 | 1 | 1 | 1 | 1 | 1 | 1 | 1 | 1 | 1 | 1 | 1 | 1 | 1 | 1 | 1 | 1 | 1 |


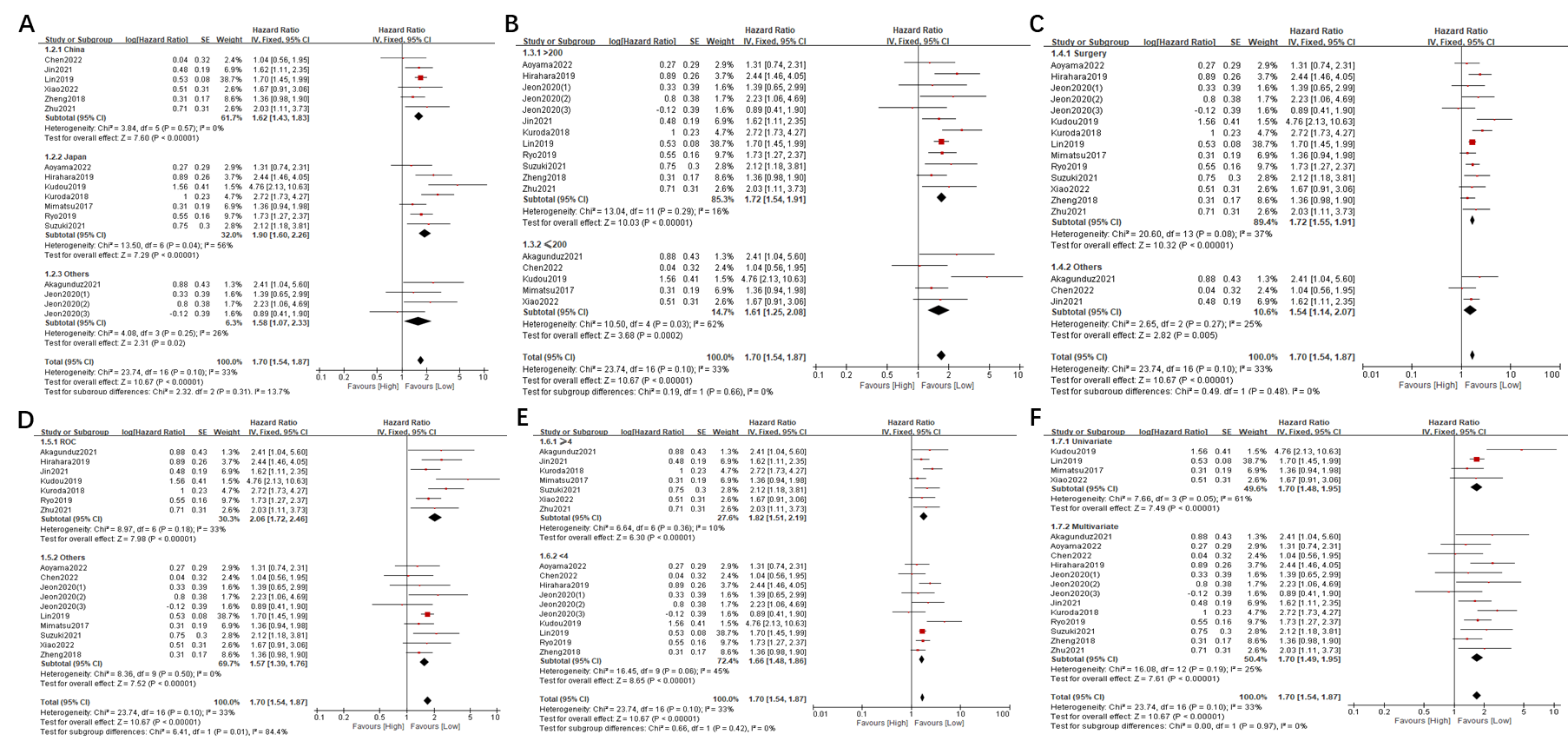


Figure S1. Forest plot of subgroup analyses assessing the relationship between CONUT and OS. A: Country (China vs. Japan vs. Others); B: Sample size (>200 vs. ≤200); C: Primary treatment (surgery vs. Others); D: Cut-off method (ROC vs. others); E: Cut-off value (≥4 vs. <4); F: Analysis method (univariate vs. multivariate).


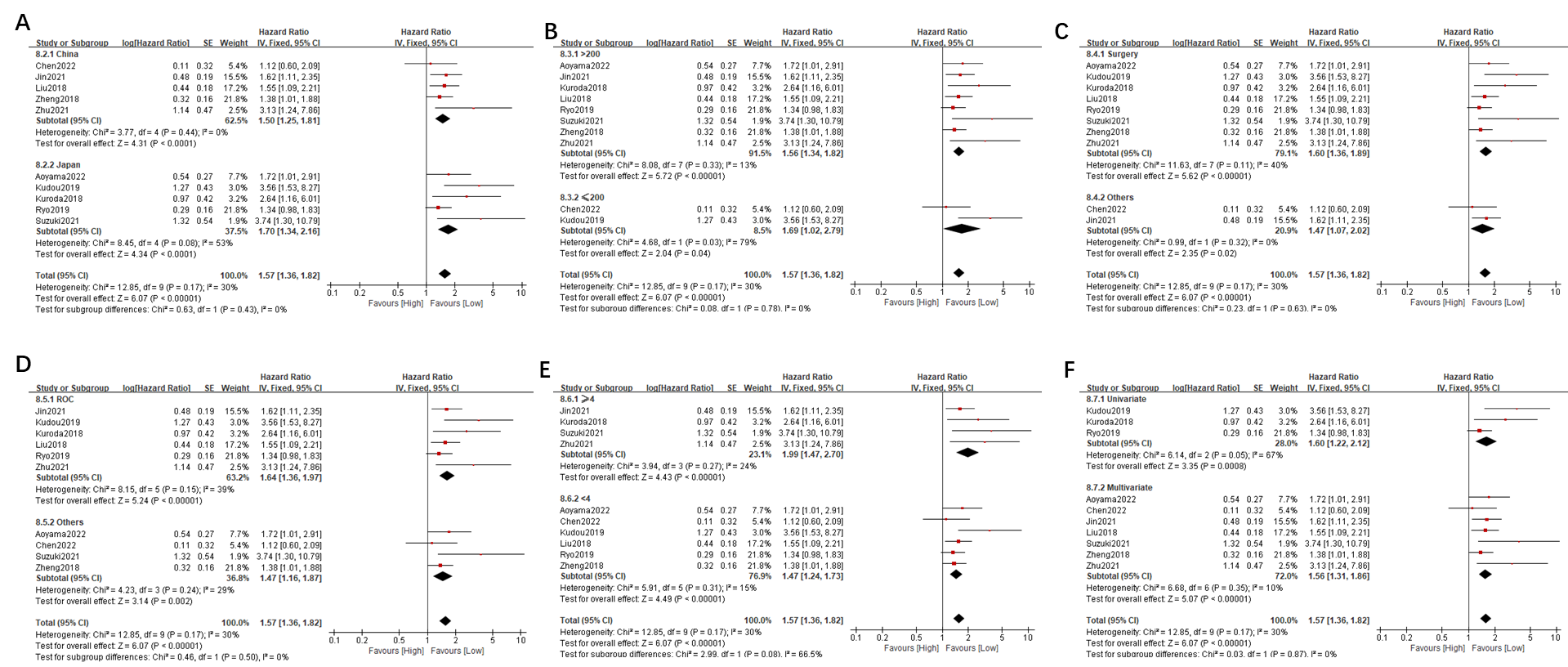


Figure S2. Forest plot of subgroup analyses assessing the relationship between CONUT and RFS. A: Country (China vs. Japan); B: Sample size (>200 vs. ≤200); C: Primary treatment (surgery vs. Others); D: Cut-off method (ROC vs. others); E: Cut-off value (≥4 vs. <4); F: Analysis method (univariate vs. multivariate).


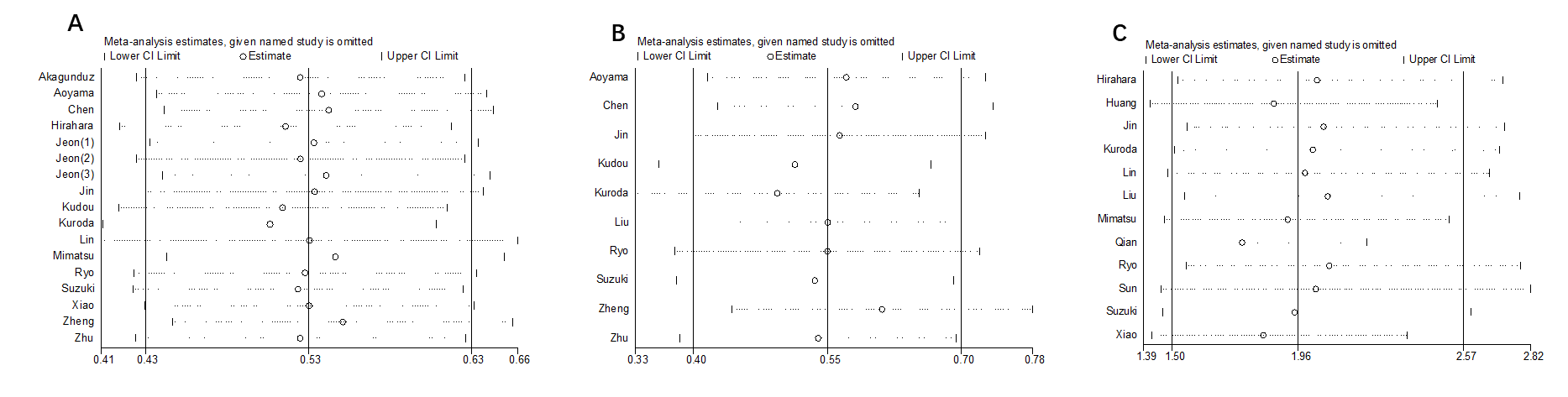


Figure S3. Sensitivity analyses assessing the relationship between CONUT and clinical outcomes including OS (A), RFS (B) and postoperative complications (C).


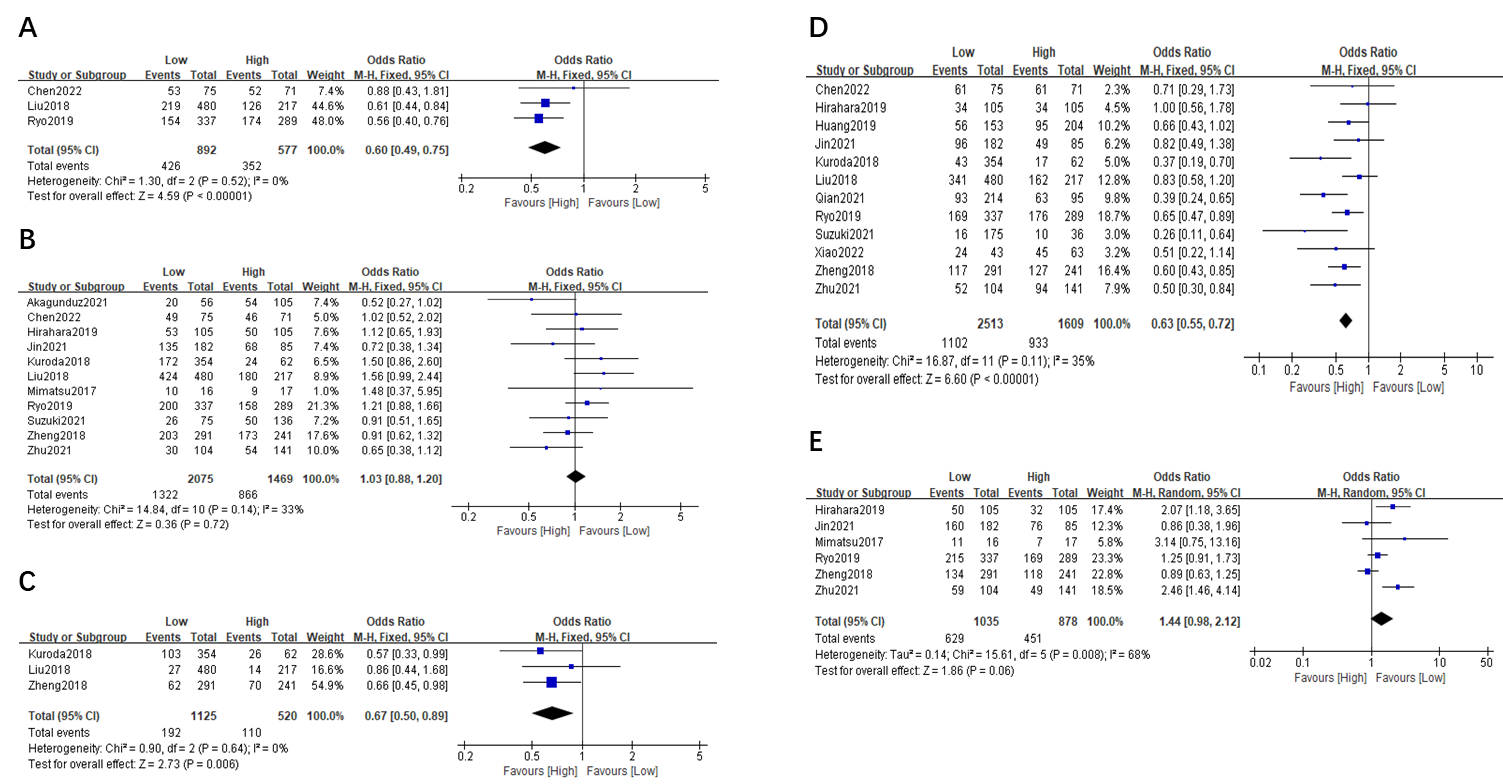


Figure S4. Forest plot of secondary outcomes assessing the relationship between CONUT and other postoperative oncological parameters, including: A, tumor size (<5cm); B, tumor differentiation (Poor differentiation); C, microvascular invasion (Yes); D, TNM stage (Stage III/IV); E, adjuvant chemotherapy (Yes).


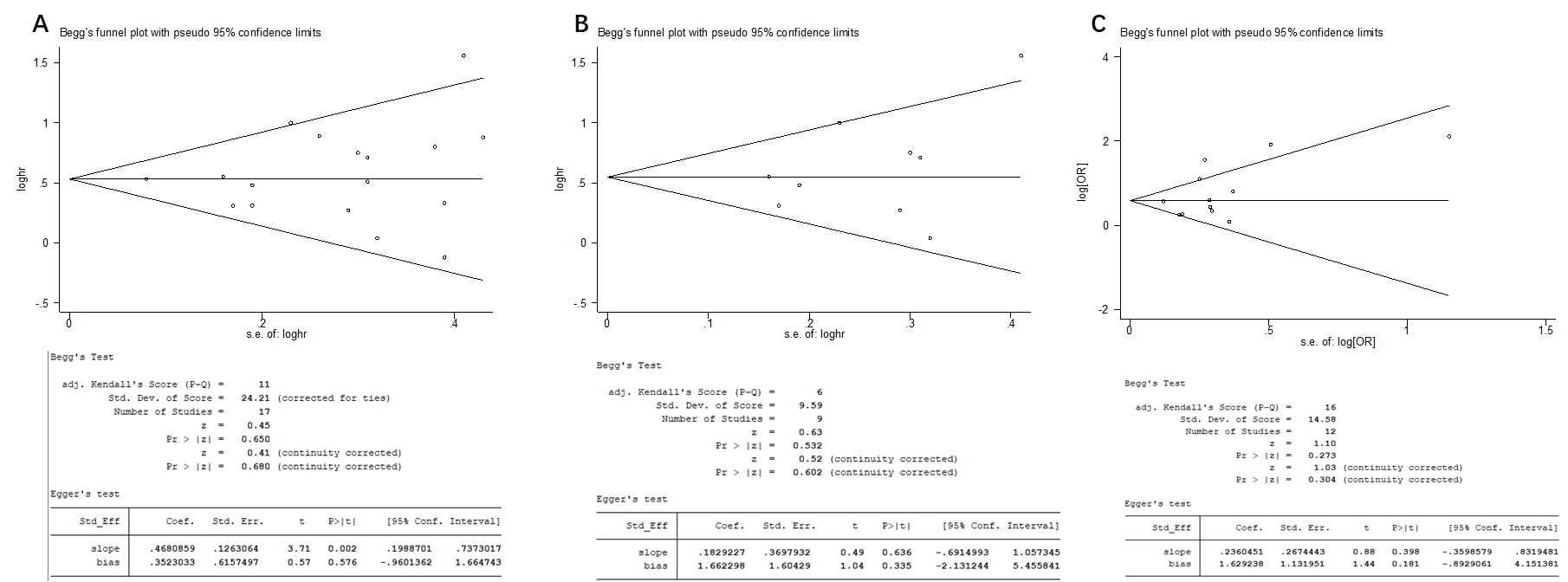


Figure S5. Begg’s funnel plot assessing publication bias between CONUT and clinical outcomes including OS (A), RFS (B) and postoperative complications (C). The Begg’s P values were 0.680,0.602 and 0.304, respectively.
